# Supplementary material for: Triplet-based similarity score for fully multilabeled trees with poly-occurring labels
Source: Bioinformatics. 2020 Jul 30;37(2):178–84. doi: 10.1093/bioinformatics/btaa676 (PMC8055217; doi:10.1093/bioinformatics/btaa676)
Supplement: btaa676_Supplementary_Data [file btaa676_supplementary_data.pdf]

Supplementary material for: Triplet-based similarity score for fully multi-labeled trees with poly-occurring labels

Simone Ciccolella[1], Giulia Bernardini[1], Luca Denti[1], Paola Bonizzoni[1], Marco Previtali[1], and Gianluca Della Vedova[1]

[1]Department of Computer Systems and Communication, University of Milano-Bicocca, Milan, Italy

1 Proof of Lemma 2

Besides the five possible configurations already listed in the proof of Lemma 1, the multi-labeled model admits four additional configurations, due to the extension of the definition of LCA of two labels. In the first three additional cases, two labels are assigned to the same node and the third one to a different one. Withouth loss of generality, suppose that a and b label the same node. These cases are: (i) $\texttt{LCA}(a, b) = \texttt{LCA}(b, c) = \texttt{LCA}(a, c)$ and the LCA is a node in $\{a, b, c\}$, (ii) $\texttt{LCA}(a, b) \neq \texttt{LCA}(b, c) = \texttt{LCA}(a, c)$ and both LCA are nodes in $\{a, b, c\}$, and (iii) $\texttt{LCA}(a, b) \neq \texttt{LCA}(b, c) = \texttt{LCA}(a, c)$ and only $\texttt{LCA}(a, b)$ is a node in $\{a, b, c\}$. The remaining case (iv) is the simplest one in which a, b, c label the same node of T, implying that $\texttt{LCA}(a, b) = \texttt{LCA}(b, c) = \texttt{LCA}(a, c)$.

Figure 2 in the main text reports cases (i) to (iv) from left to right.

2 Experimental comparison of MP3

We show here an experimental comparison of the different versions of MP3, demonstrating the reason we decided to use MP3 $_\sigma$ as our default measure. We show in Figure S1 (a) that MP3 $_\sigma$ combines the best aspect of MP3 $_\cap$ and MP3 $_\cup$ while MP3 $_G$ is, as expected, the average of the two. The same result can be seen in Figure S1 (b), while Figure S1 (c) display the effect of the sigmoid, where as the trees become less similar the value move towards the union.

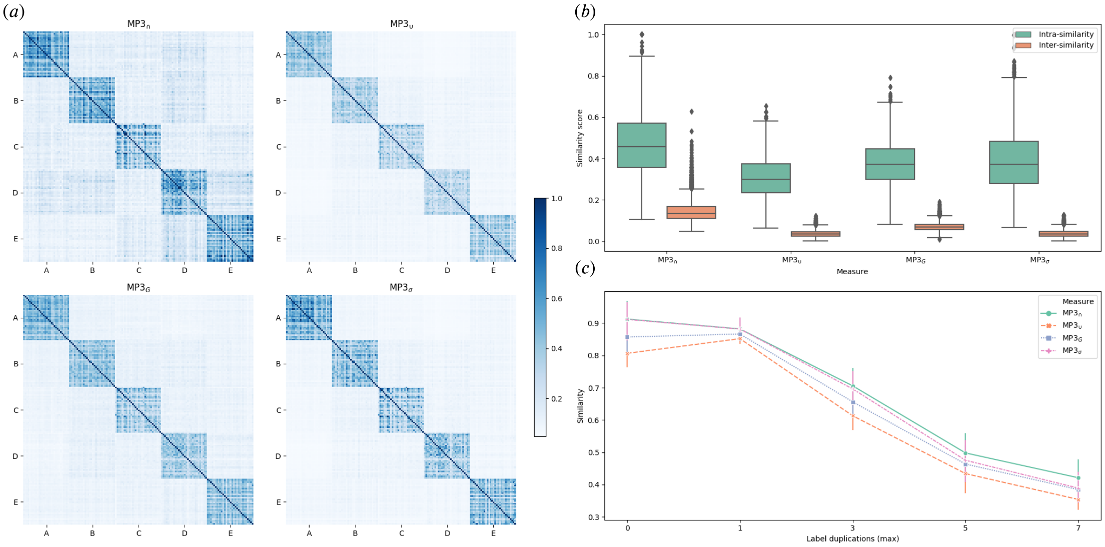

Figure S1: (a) Heatmaps displaying the scores between all the 150 simulate trees from the second experimental setting. (b) Distribution of the similarities between the trees in the same class (Intra-similarity) and in different classes (Inter-similarity) for the 5 classes. (c) Effect of label duplication on the similarity scores. Similarities are the average of 15 trees generate from the same base with the specified value of maximum duplication from the previous experiment.

3 Effect of label sliding

We present a comparison of the measure in the case of a label sliding from left to right on the lowest level of a binary tree. The trees are compared against the first tree T_0. When the label slide to the same subtree no difference is found for all measure, as expected. On the other hand we show a higher decrease in similarity for `MP3` with respect to the other measure as the label slides. Interestingly for the last two trees CASet and DISC collapse to the same value showing no difference between the two. MLTD express very little difference between the last four trees.

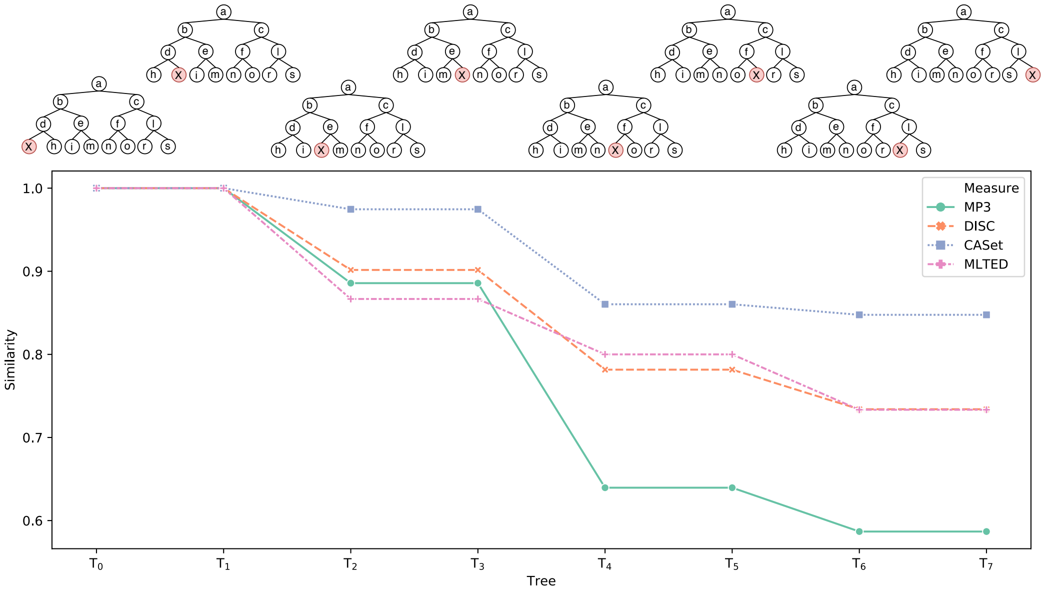

Figure S2: Effect of a label sliding from left to right on the lowest level of a binary tree.

4 Base tree for poly-occurring label experiment

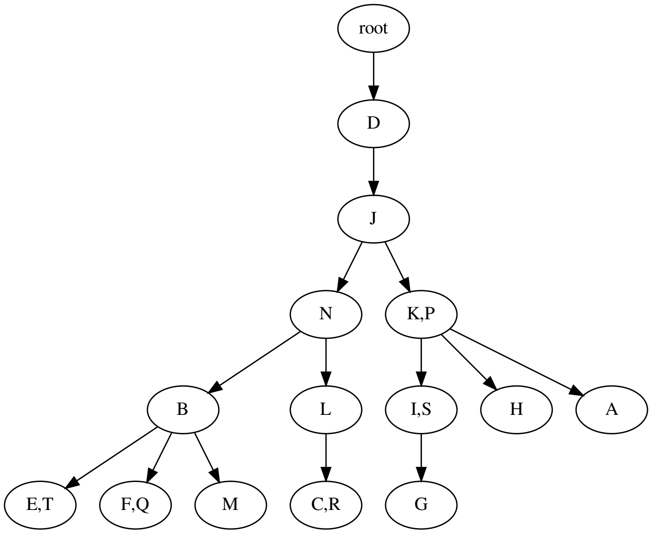

Figure S3: Base tree used for evaluating the effect of poly-occurring labels on the similarity scores.

5 Base trees for Exp1 and Exp2

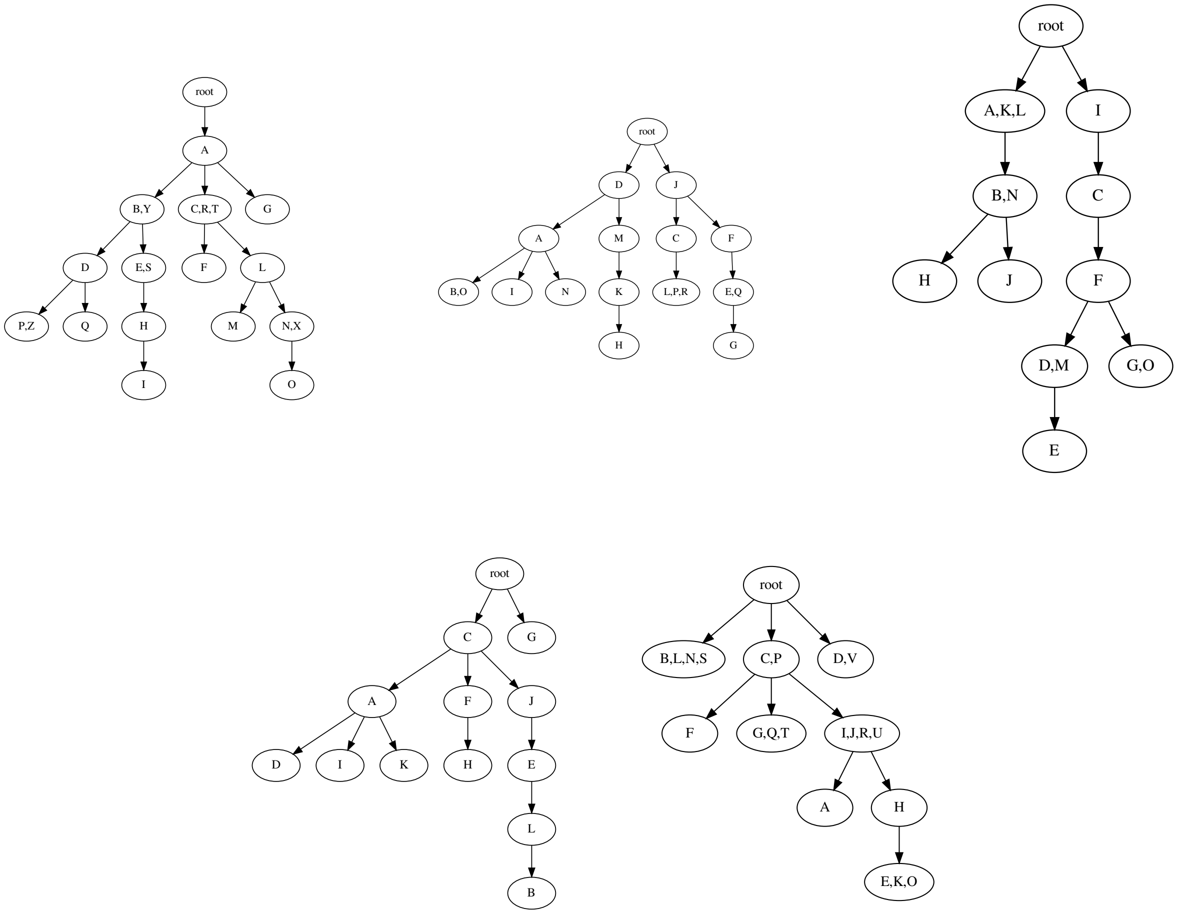

Figure S4: Base trees used in Experiments 1 and 2.

6 Base trees for Clustering experiment

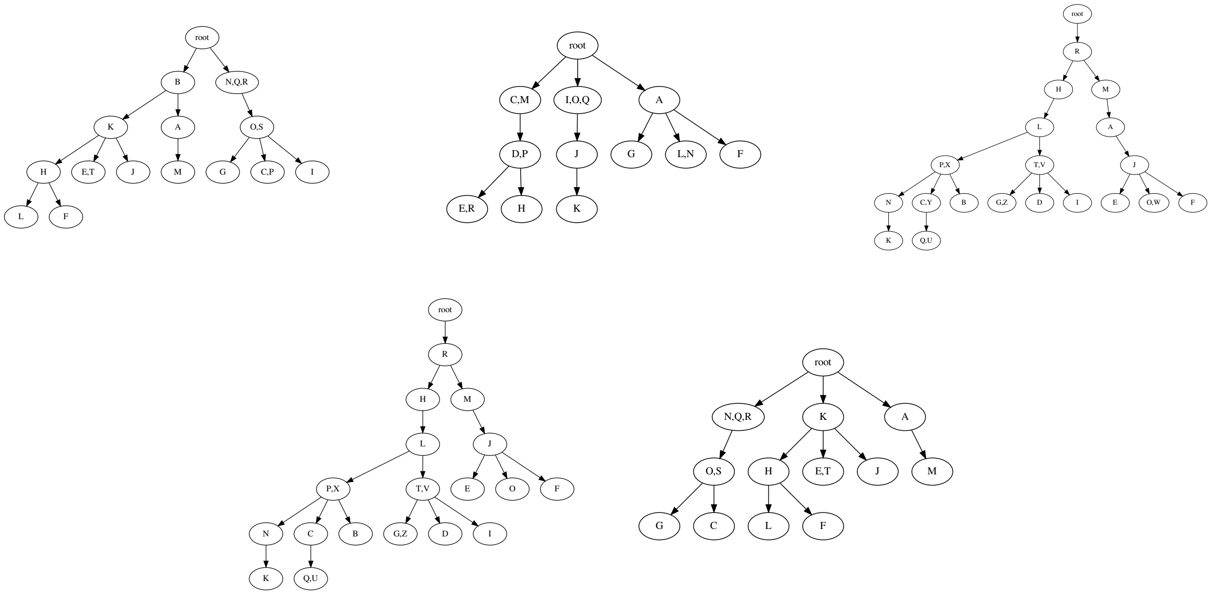

Figure S5: Base trees used in the clustering experiment. Tree 4 is a perturbation of Tree 3 and Tree 5 is a perturbation of Tree 1.

7 Trees for real data experiment

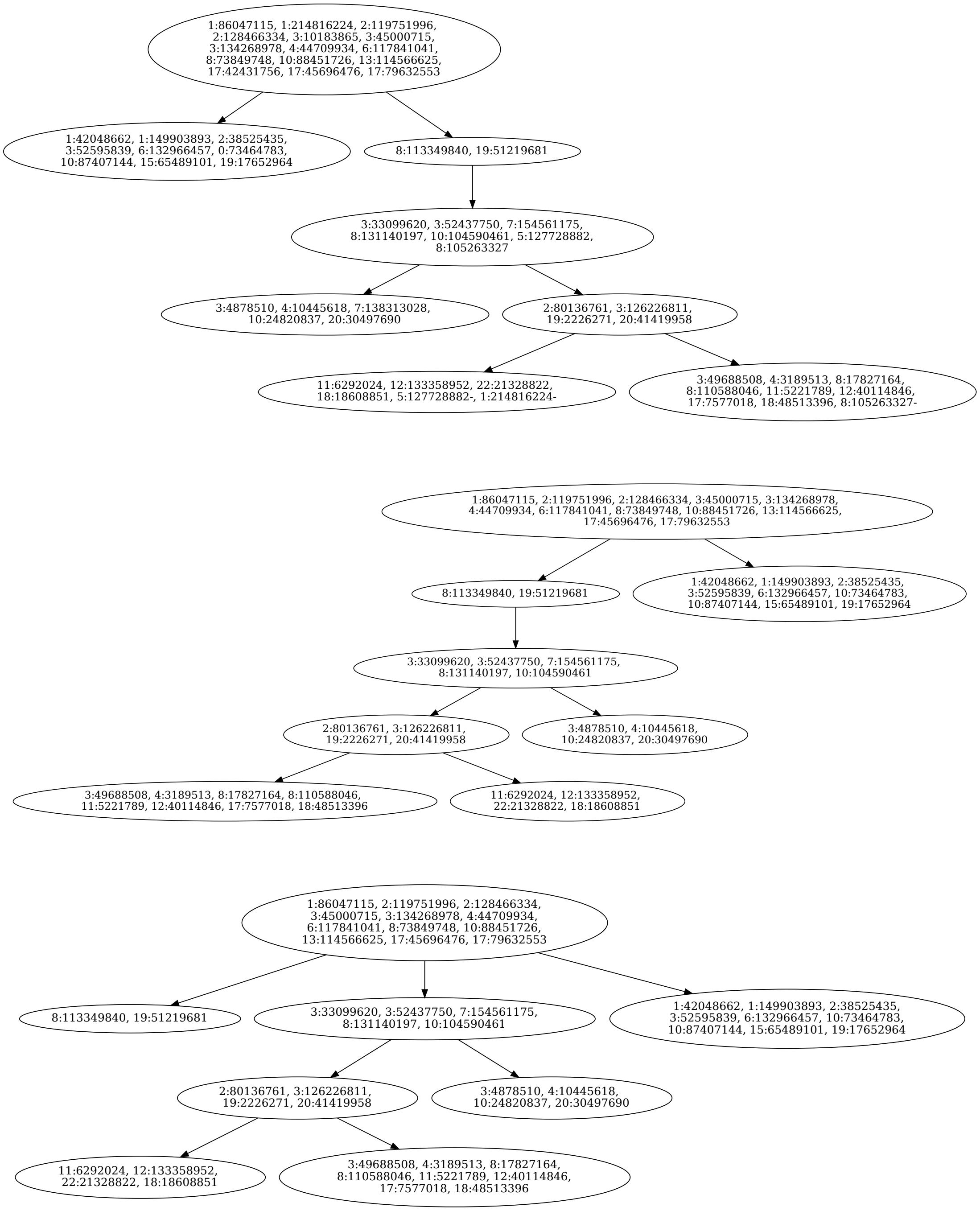

Figure S6: Trees used in the experiment on real data from Gerlinger et al. (2014). The upper tree is the base tree proposed in Gerlinger et al. (2014) for patient RMH002, the second one is the tree inferred by LICHeE, and the last one is the tree inferred by MIPUP (ipd parameter). We crafted the first tree starting from the supplementary material of the corresponding paper whereas we built the other two considering the trees reported in the MIPUP github repository.

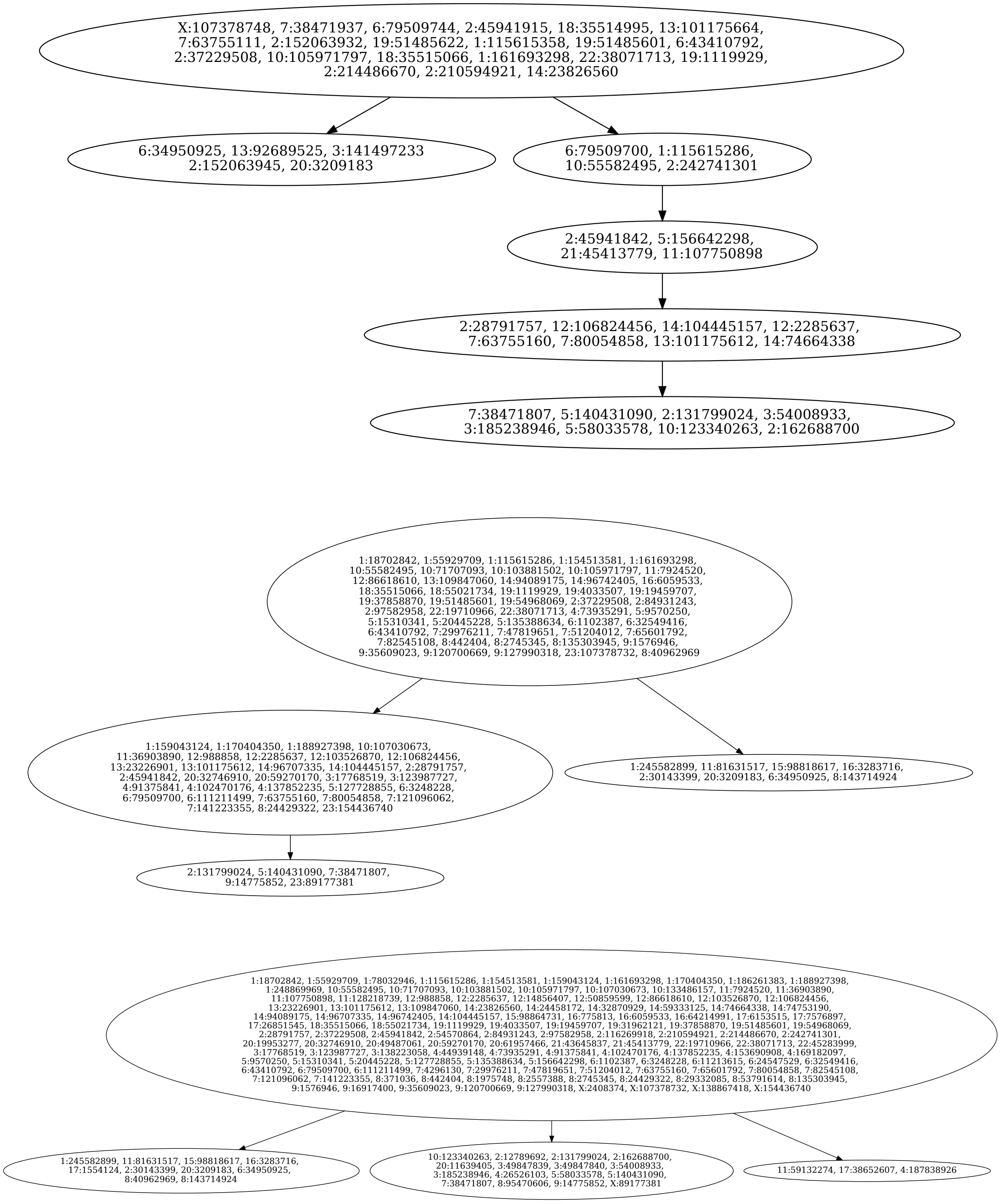

Figure S7: Trees used in the experiment on real data from Eirew et al. (2015). The upper tree is the base tree proposed in Eirew et al. (2015) for case SA501, the second one is the tree inferred by LICHeE, and the last one is the tree inferred by MIPUP. We obtained these trees from the supplementary material of DiNardo et al. (2019).

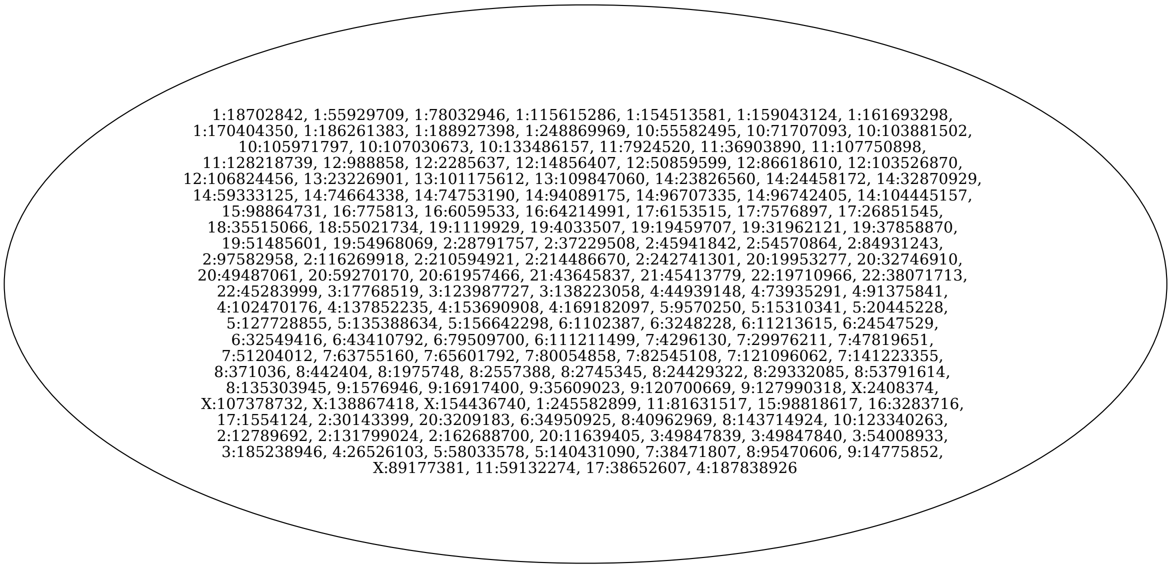

Figure S8: Edge case tree used in the experiment on real data from Eirew et al. (2015). We obtained such a tree by collapsing all nodes of the MIPUP's tree from Figure S7 in a single node.

8 Example of computation of MP3

To better understand how to compute MP3, in this section we report a detailed example on trees with repeated labels.

In Figure S9 we present two trees: the tree on the left, that we will refer to as Tree A, is composed of six nodes and does not include any repeated label, whereas the tree on the right, that we will refer to as Tree B, is composed of six nodes and only five distinct labels.

In Figure S10 we report the 20 possible MTTs of Tree A and in Figure S11 we report the 20 possible MTTs of Tree B.

Note that the MTTs of Tree A are unique, whereas the MTTs of Tree B are not. Indeed, since the label c appears twice, two MTTs of Tree B have the same labels (a, c, and f) and, by chance, the same shape (top right of Figure S11). Notice that the same MTT is in the multiset of MTTs of Tree A (top right of Figure S10) but appears only once.

In Figure S12 we report the set of MTTs shared between Tree A and Tree B. This set is composed of seven MTTs and note that the MTT of the triplet (a, c, f) appears only once. This means that the MTT of that triplet will appear twice in $\mathtt{M}_B(a, c, f)$, once in the $\mathtt{M}_A(a, c, f) \sqcap \mathtt{M}_B(a, c, f)$, and once $M_B(a, c, f) \setminus M_A(a, c, f)$.

Overall, we have the following (we refer the reader to Section 2.2 of the main document for the notation):

- $|\lambda(A)| = 6$
- $|\lambda(B)| = 5$
- $|I| = 10$, *i.e.*, the number of the triplets over the multiset $\{a, b, c, e, f\}$
- $|J| = 35$, *i.e.*, the number of the triplets over the multiset $\{a, b, c, c, d, e, f\}$

- $\texttt{MP3}_\cap = 7/10 = 0.7$
- $\texttt{MP3}_\cup = 7/35 = 0.2$
- $\texttt{MP3}_\sigma = 0.2 + \sigma(0.7) \times \min\{0.7 - 0.2, 0.2\} \simeq 0.22384$

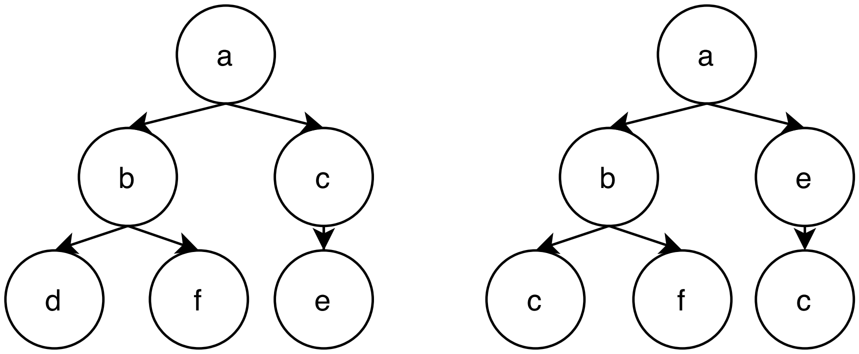

Figure S9: Two tumor progression trees. Tree A (left) is composed of six nodes and six different labels, whereas Tree B (right) is composed of 6 nodes and five distinct labels.

References

Zach DiNardo, Kiran Tomlinson, Anna Ritz, and Layla Oesper. Distance measures for tumor evolutionary trees. *Bioinformatics*, 11 2019. ISSN 1367-4803. doi: 10.1093/bioinformatics/btz869. URL `https://doi.org/10.1093/bioinformatics/btz869`. btz869.

Peter Eirew, Adi Steif, Jaswinder Khattra, Gavin Ha, Damian Yap, Hossein Farahani, Karen Gelmon, Stephen Chia, Colin Mar, Adrian Wan, et al. Dynamics of genomic clones in breast cancer patient xenografts at single-cell resolution. *Nature*, 518(7539):422–426, 2015.

Marco Gerlinger, Stuart Horswell, James Larkin, Andrew J Rowan, Max P Salm, Ignacio Varela, Rosalie Fisher, Nicholas McGranahan, Nicholas Matthews, Claudio R Santos, et al. Genomic architecture and evolution of clear cell renal cell carcinomas defined by multiregion sequencing. *Nature genetics*, 46(3):225, 2014.

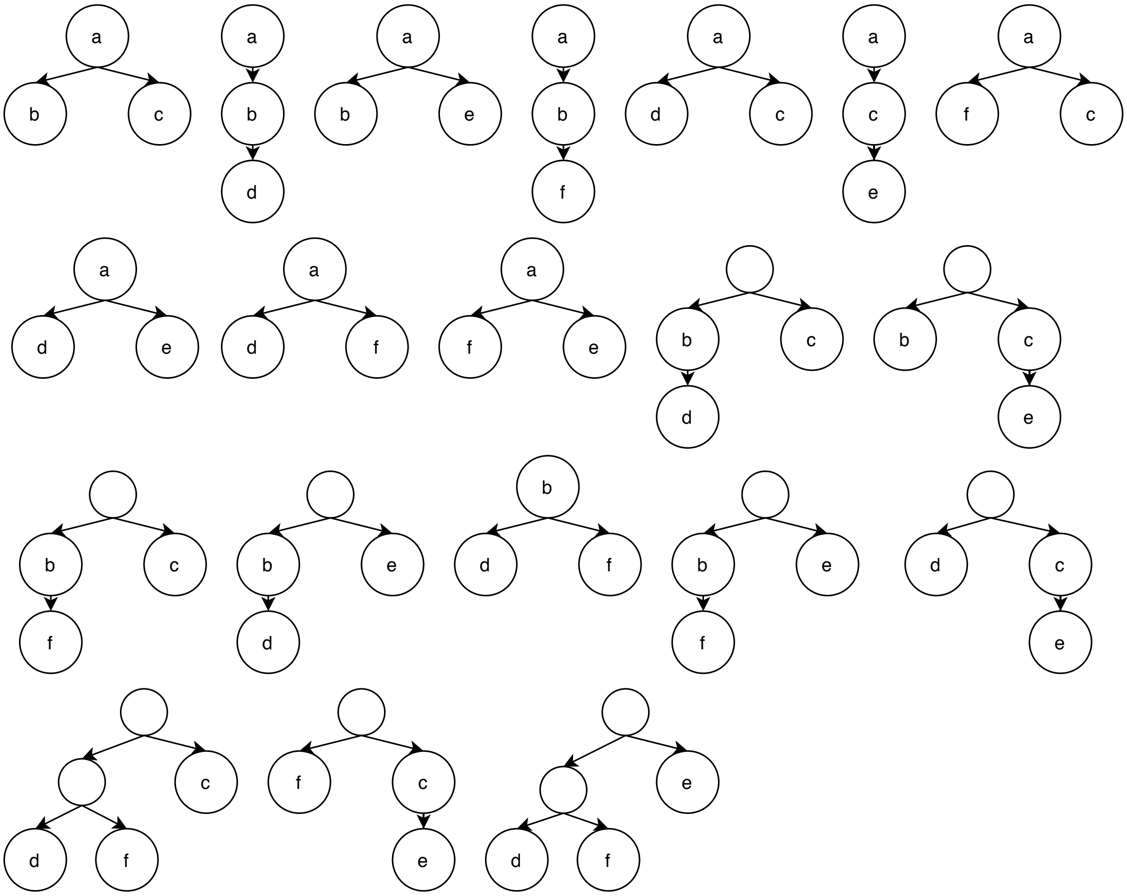

Figure S10: Minimal tree topology of each triplet of nodes in Tree A.

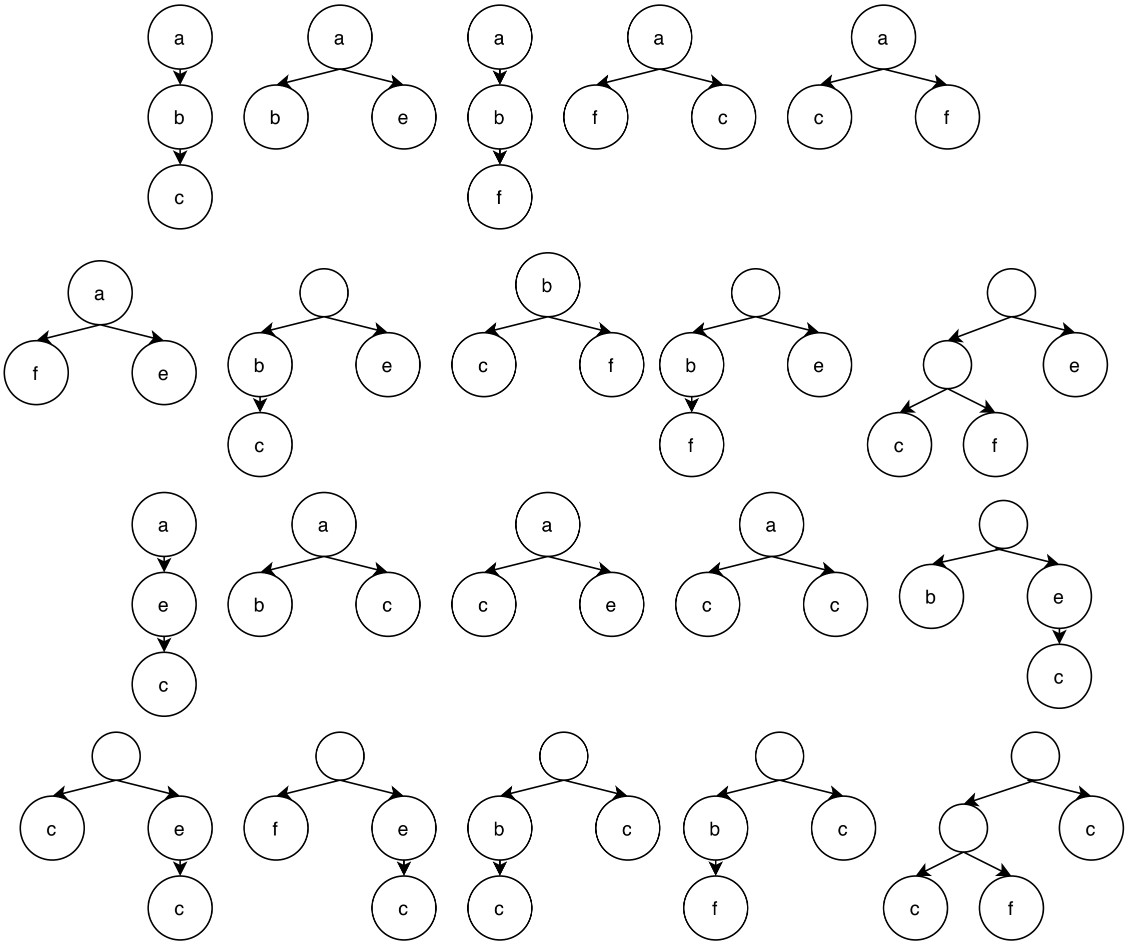

Figure S11: Minimal tree topology of each triplet of nodes in Tree B.

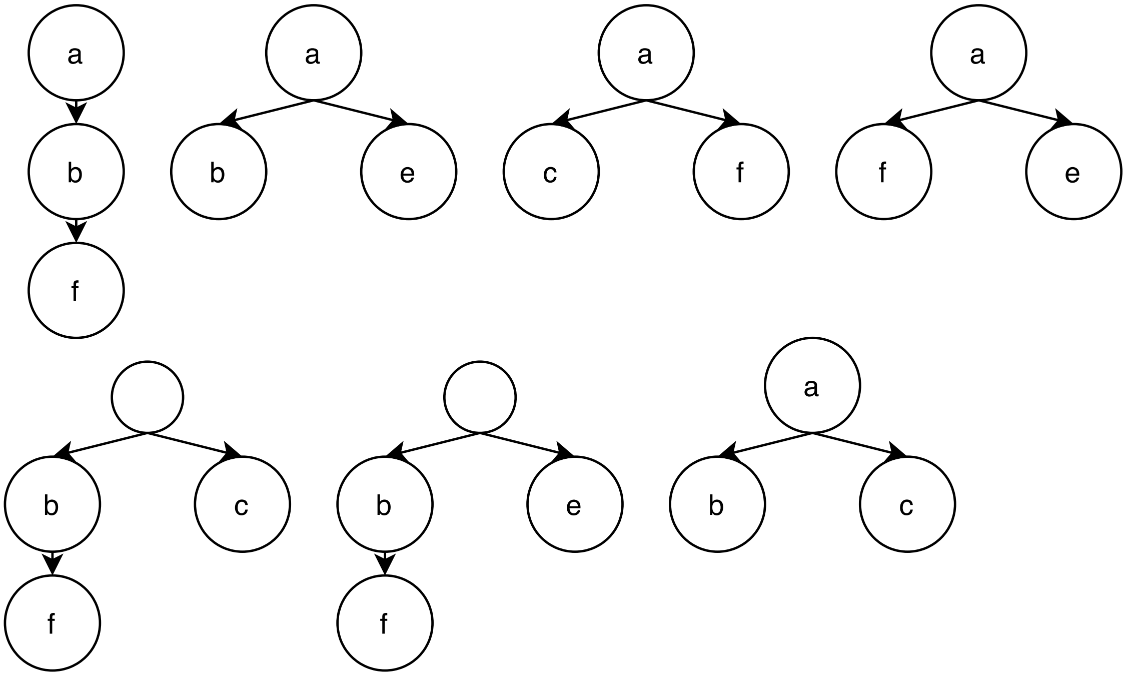

Figure S12: Shared Minimal tree topologies shared between Tree A and Tree B. Note that the MTT of the triplet (a, c, f) appears only once.
